# Decreased plasma concentrations of apolipoprotein M in sepsis and systemic inflammatory response syndromes

**DOI:** 10.1186/cc11305

**Published:** 2012-04-18

**Authors:** Sunil B Kumaraswamy, Adam Linder, Per Åkesson, Björn Dahlbäck

**Affiliations:** 1 Department of Laboratory Medicine, Division of Clinical Chemistry, Lund University, Skåne University Hospital, Entrance 46, Malmö, SE-20502, Sweden; 2 Department of Clinical Sciences, Division of Infection Medicine, Lund University, Skåne University Hospital, Tornav 10, Lund, SE-22184, Sweden

## Abstract

**Introduction:**

Apolipoprotein M (apoM) is present in 5% of high-density lipoprotein (HDL) particles in plasma. It is a carrier of sphingosine-1-phosphate (S1P), which is important for vascular barrier protection. The aim was to determine the plasma concentrations of apoM during sepsis and systemic inflammatory response syndrome (SIRS) and correlate them to levels of apolipoprotein A-I (apoA1), apolipoprotein B (apoB), HDL-, and low-density lipoprotein (LDL)-cholesterol.

**Methods:**

Plasma samples from patients with (1), severe sepsis with shock (*n *= 26); (2), severe sepsis without shock (*n *= 44); (3), sepsis (*n *= 100); (4), infections without SIRS (*n *= 43); and (5) SIRS without infection (*n *= 20) were analyzed. The concentrations of apoM, apoA1, and apoB were measured with enzyme-linked immunosorbent assays (ELISAs). Total, HDL-, and LDL-cholesterol concentrations were measured with a commercial HDL/LDL cholesterol test.

**Results:**

ApoM concentrations correlated negatively to acute-phase markers. Thus, apoM behaved as a negative acute-phase protein. Decreased values were observed in all patient groups (*P *< 0.0001), with the most drastic decreases observed in the severely sick patients. ApoM levels correlated strongly to those of apoA1, apoB, HDL, and LDL cholesterol. The HDL and LDL cholesterol levels were low in all patient groups, as compared with controls (*P *< 0.0001), in particular, HDL cholesterol. ApoA1 and apoB concentrations were low only in the more severely affected patients.

**Conclusions:**

During sepsis and SIRS, the plasma concentrations of apoM decrease dramatically, the degree of decrease reflecting the severity of the disease. As a carrier for barrier-protective S1P in HDL, the decrease in apoM could contribute to the increased vascular leakage observed in sepsis and SIRS.

## Introduction

Apolipoprotein M (25 kDa) is associated mainly with HDL and, to a minor extent, with apoB-containing lipoproteins [1,2]. It is anchored to the lipoprotein particles with a retained signal peptide [1,3,4]. Its normal plasma concentration is approximately 23 mg/L (0.9 μ*M*). In normal plasma, it has been estimated that around 5% of HDL particles contain an apoM molecule [5]. The plasma concentration of apoM correlates with the plasma levels of total, HDL, and LDL cholesterol [6]. ApoM is a member of the lipocalin protein family, a group of proteins with a characteristic coffee filter-like structure and a hydrophobic binding pocket [2,7]. The elucidation of the three-dimensional structure revealed that apoM has the ability to bind sphingosine-1-phospate (S1P), an important bioactive lipid mediator known to be associated with HDL [8-13]. In plasma, approximately 60% of S1P is bound to HDL, 5% to LDL, and the rest to albumin. ApoM is the protein in HDL that binds S1P, and it was recently shown that endothelium-protective effects of S1P are provided by S1P bound to the HDL-associated apoM [8].

S1P can activate five different G-protein-coupled receptors present on many different cells, and it is involved in regulation of many cellular functions, including migration, angiogenesis, adhesion, inflammation, and rearrangement of the cytoskeleton [10-13]. The concentration of S1P is higher in blood and lymph as compared with interstitial fluid (for example, in the lymphoid organs), and the S1P gradient is important for the trafficking of immune cells (for example, the egress of lymphocytes from the thymus and other lymphoid organs). S1P is also important for the cell-to-cell contacts of endothelium that regulate the basal barrier function and inflammation-induced vascular leak. In this context, it is noteworthy that the expression of apoM in both liver and kidney decreases during inflammation, which results in decreased plasma levels of apoM [14].

The acute-phase response (APR) can be triggered by infections or other causes of inflammation, such as tissue trauma, ischemic necrosis, and malignancies [15]. The APR is characterized by increased concentrations of certain plasma proteins (for example, serum amyloid A (SAA), C-reactive protein (CRP), α_1_-antitrypsin, and fibrinogen), whereas others decrease, such as albumin and transferrin. These changes are caused by the influence of inflammatory cytokines on transcription factors that control the rate of synthesis of the different proteins. The lipid and lipoprotein profiles are dramatically changed during the APR [16-19]. The plasma concentrations of total cholesterol, HDL, and LDL cholesterol decrease, whereas triglyceride and very low-density lipoprotein (VLDL) levels increase. The plasma levels of apoA1 decrease during APR, and the protein composition of acute-phase HDL changes drastically, SAA becoming the dominating protein. HDL is part of the innate immune defense, being able to bind and neutralize lipopolysaccharide (LPS), an activity shared with the other lipoproteins. The LPS-binding activity of the lipoproteins increases during the acute-phase reaction because of the increased levels of LPS-binding protein (LBP) [20,21]. SAA-containing HDL has a higher elimination rate than normal HDL, which adds to the efficiency of LPS elimination from circulation. Other important changes in HDL that affect the function of HDL include decreased levels of lecithin cholesterol acyl transferase (LCAT), cholesterol ester transfer protein (CETP), and the antioxidant enzyme paraoxonase 1 (PON-1), whereas LBP and the activities of phospholipid transfer protein (PLTP) and platelet-activating factor acetylhydrolase (PAF-AH) increase. As a result of these changes, the reverse cholesterol transport capacity of HDL decreases, and HDL loses its antioxidant and antiinflammatory properties [16-19,22].

Sepsis, which causes a severe form of APR and systemic inflammatory response syndrome (SIRS), is a leading cause of mortality in noncoronary intensive care units [23-26]. The yearly incidence is approximately three cases per 1,000 individuals, and the mortality is high. The severe forms of sepsis, which cause septic shock, result in severely decreased plasma levels of HDL cholesterol, apoA-1, apoC1, and CETP, and these factors have been suggested to be independent predictors of survival [27-32].

ApoM is a negative acute-phase protein that decreases during infection and inflammation [14]. In a pilot study including six patients with sepsis, the plasma concentration of apoM, estimated by semiquantitative Western blotting, was found to be decreased to around 60%, as compared with the apoM levels after recovery [14]. This observation called for a larger study of apoM in sepsis and SIRS. The aim of this study was to investigate the apoM concentration in plasma in a large cohort of patients with sepsis and SIRS. We report that the apoM plasma levels were drastically decreased in all patients, and a strong relation was seen between the decrease of apoM and the severity of the disease.

## Materials and methods

### Study population

The study cohort that included 232 patients was previously described in detail [33]. In brief, the patients were recruited in a prospective study at the Clinic for Infectious Diseases, University Hospital, Lund, Sweden. The criteria for inclusion were fever (≥ 38°C) and a suspected infection, and only patients older than 18 years were included. Citrated plasma was collected within 12 hours after admission to the hospital. Healthy controls from the local blood bank (*n *= 97) served as controls. Patients and controls were not fasting at the time of blood collection.

The patients were judged by the SIRS criteria (that is, body temperature ≥ 38°C, white blood cell count (WBC) > 12 × 10^9^/L or < 4 × 10^9^/L, pulse rate > 90/minute, and respiratory rate > 20/minute [34], by significant hypotension, with a systolic blood pressure of < 90 mm Hg or a decrease of > 40 mm Hg from baseline, and by the presence or absence of organ failure, as well as the final diagnosis. Based on the criteria, the patients were divided into the following groups: (1), severe sepsis with shock (*n *= 26); (2), severe sepsis without shock (*n *= 44); (3), sepsis (*n *= 100); (4), infections without SIRS (*n *= 43); and (5) SIRS without infection (*n *= 20) [33].

Severe sepsis was defined as an infectious disease and at least two SIRS criteria, and the presence or development of hypotension and/or organ failure within 24 hours of the collection of the blood samples [33]. Septic shock was severe sepsis with hypotension that required vasopressor support or a persistent hypotension for more than 1 hour despite adequate fluid resuscitation. Sepsis was defined as an infectious disease, at least two SIRS criteria, but no presence or development of organ failure. Infection was defined as an infectious disease without SIRS. SIRS without infections had at least two SIRS criteria and one of the following diseases: systemic vasculitis, cardiac failure, gastrointestinal bleeding, pulmonary embolism, corticosteroid deficiency due to hypopituitarism, dehydration, or urine retention [33].

The ethics committee of Lund University approved the project protocol (number 790/2005), and informed consent was obtained from all patients or their close relatives.

### Analysis of apoA1, apoB, and apoM

The ApoM ELISA was described previously [6]. In brief, 96-well Costar plates (Corning, Inc., Lowell, MA, USA) were coated with a catching monoclonal apoM antibody (mAb 58) before blocking with a quenching solution. The samples were diluted in detergent-containing buffer and incubated overnight. The bound apoM was detected by using a biotinylated secondary apoM antibody (mAb 42) in combination with streptavidin-avidin-horseradish peroxidase and 1,2-phenylenediamine dihydrochloride (Dako, Glostrup, Denmark). The 490-nm absorbance was measured and compared with a plasma standard curve having known amounts of apoM.

ApoA1 and apoB also were measured with ELISA assays, which were similar in design to the apoM ELISA. For apoA1, a polyclonal antibody against apoA1 (Q0496; Dako) was used as catcher, whereas an apoA1 monoclonal antibody (mAb25) made in the laboratory was used as the detecting antibody. For apoB, a polyclonal antibody directed against apoB (Q0497; Dako) was used as a catcher, and an apoB monoclonal antibody (ACC-250319; Nordic Biosite, Täby, Sweden) was used as a detecting antibody.

### Analysis of HDL, LDL, and total cholesterol

Plasma concentrations of HDL cholesterol, LDL cholesterol, and total cholesterol were measured with the combined HDL/LDL cholesterol kit (EHDL-100; Bioassay Systems, Hayward, CA, USA). The kit is based on a polyethylene glycol (PEG) precipitation in which HDL and LDL/VLDL are separated, and the cholesterol concentrations then were determined by using cholesterol esterase/cholesterol dehydrogenase reagent.

### Statistical analysis

Nonparametric tests were used throughout the study. The Mann-Whitney *U *test was used for evaluating the difference between different groups, and the Spearman rank correlation coefficient, for evaluating correlations. For all tests, *P *< 0.05 was considered significant. Graphpad Prism 4.0 (Graphpad Software, La Jolla, CA, USA) was used for statistics.

## Results

### Patients

The study included 232 patients with fever and suspected infection, of whom 26 were found to have severe sepsis with shock; 44 had severe sepsis without shock; 99 had sepsis; 43 had infections without SIRS; and 20 had SIRS without infections. Patient demographic details and diagnoses were presented elsewhere [33]. Urinary tract infections and pneumonia were common, in particular in the sepsis groups, whereas upper respiratory infections dominated in patients without SIRS. The patients with noninfectious SIRS had various diseases (for example, vasculitis, cardiac failure, gastrointestinal bleeding, pulmonary embolism, or pancreatitis). In the whole patient population, the mortality was 3.4%; in the severe-sepsis groups, it was 10%, and in the severe-sepsis group with shock, it was 19%.

### HDL, LDL, and total cholesterol plasma concentrations

**Figure 1:**
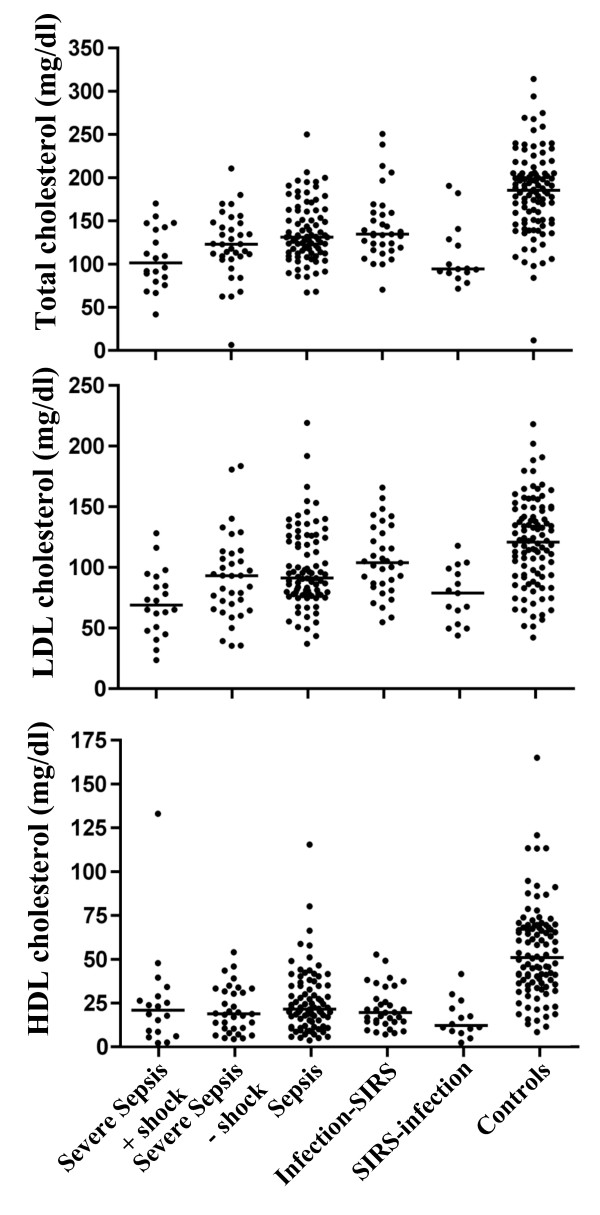
**Plasma concentrations of total, low-density-lipoprotein (LDL), and high-density-lipoprotein (HDL) cholesterol in the patients**. The bars represent the median values (detailed in Table 1).

The plasma concentrations of total cholesterol and HDL and LDL cholesterol demonstrated similar patterns of variation between the five patient groups, with the most-pronounced decreases seen in patients with severe sepsis with shock, followed by the severe sepsis group without shock, and the patients with SIRS without infections (Figure 1). In all patient groups, the levels of total, LDL, and HDL cholesterol were highly significantly lower (*P *< 0.001) than in controls. In the most severely affected groups, the median cholesterol levels were > 50% of the corresponding levels of the controls (Table 1). It was noteworthy that in the group with severely sick patients with SIRS but without infections, the cholesterol levels were equally low or lower than the cholesterol levels of the most severely affected sepsis group.

**Table 1:** Concentrations of apoM, apoA1, apoB, HDL cholesterol, LDL cholesterol, total cholesterol, and CRP in patients and controls

	apoM (μ*M*)	apoA1 (g/L)	apoB (g/L)	**HDL chol**. (mg/dl)	**LDL chol**. (mg/dl)	**Total chol**. (mg/dl)	CRP (mg/L)
Severe sepsis + shock	0.36^c^ (0.13-0.84)	1.51^c^ (0.48-3.42)	0.57^c^ (0.23-1.70)	21^c^ (2.4-131)	69^c^ (23-129)	102^c^ (42-170)	180 (11-532)
Severe sepsis - shock	0.51^c^ (0.20-0.91)	1.83^a^ (0.16-4.18)	0.74 (0.09-2.30)	19^c^ (4.6-53)	93^c^ (34-184)	123^c^ (7-211)	155 (6-564)
Sepsis	0.64^c^ (0.22-1.10)	2.12 (0.76-4.49)	0.88 (0.22-2.06)	21^c^ (3.9-114)	91^c^ (37-220)	131^c^ (67-250)	112 (5-473)
Infection/sepsis	0.73^c^ (0.33-0.93)	2.40 (1.00-4.76)	1.01 (0.47-1.90)	19^c^ (7.2-52)	104^c^ (55-166)	135^c^ (70-250)	37 (1-192)
SIRS infection	0.51^c^ (0.13-0.99)	1.30^c^ (0.69-3.96)	0.92 (0.22-1.54)	12^c^ (2.6-41)	79^c^ (44-118)	94^c^ (72-191)	84 (1-468)
Controls	0.89 (0.65-1.48)	2.18 (1.15-4.66)	0.98 (0.36-2.22)	50 (8.6-163)	121 (42-219)	185 (12-314)	NA

The medians and ranges are given. The CRP levels for controls were not analyzed (NA); the local normal range for CRP was < 3 mg/L. The asterisks indicate the level of significance in difference between patient groups and controls: ^a^p < 0.05; ^c^p < 0.001.

### ApoA1, ApoB, and apoM plasma concentrations

**Figure 2:**
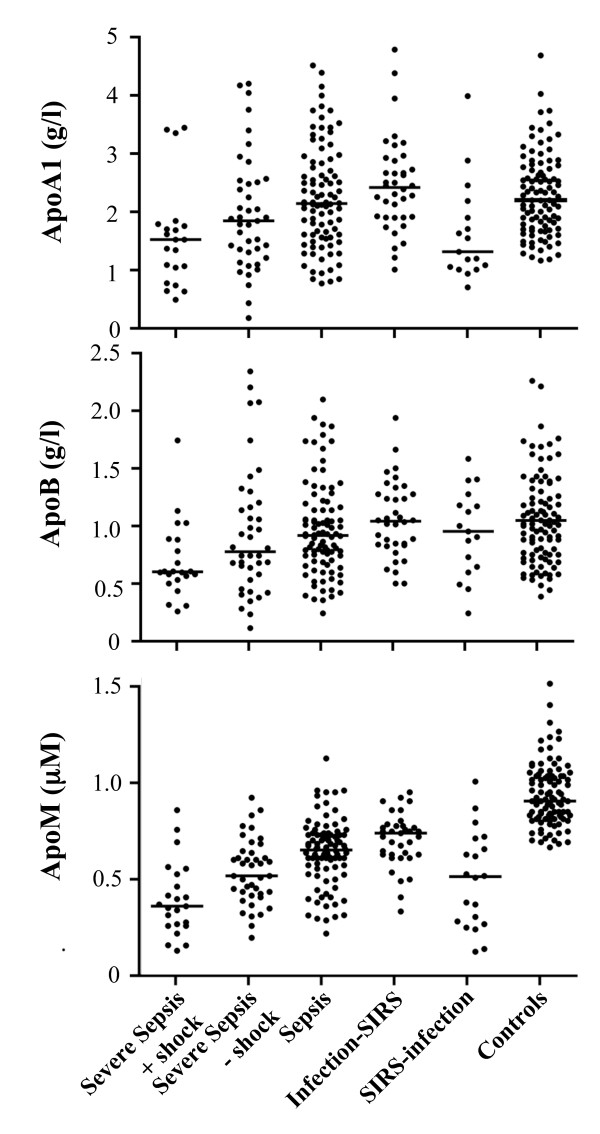
**Plasma concentrations of apolipoprotein (apo)AI, apoB, and apoM in the patients**. The bars represent the median values (detailed in Table 1).

The plasma concentrations of apoA1, apoB, and apoM demonstrated a similar pattern of variation between the five patient groups (Figure 2). The most pronounced decreases were observed in patients with severe sepsis with shock, followed by the severe sepsis group without shock and the patients with SIRS without infections. ApoA1 levels were lowest in patients with severe sepsis with shock and also in the SIRS group without infections, and both groups were highly significantly different from the control group (*P *< 0.001) (Table 1). The apoA1 in the group having severe sepsis minus shock differed less from the apoA1 in controls, but the difference was still significant (*P *= 0.0127). ApoB levels were less affected and was significantly lower than controls only in patients with severe sepsis and shock (*P *< 0.001). The plasma levels of apoM in the five patient groups were all highly significantly lower than the apoM in controls (*P *< 0.001 for all groups). In the three sepsis groups, the lowest apoM levels were found in the group with severe sepsis and shock, followed by the severe sepsis group without shock and the sepsis group. The group with SIRS without infection also demonstrated very low apoM levels. ApoM levels in the patient group with infections without SIRS were also significantly lower than apoM levels in controls. Thus, among the apolipoproteins tested, apoM demonstrated the largest differences compared with the controls.

### Correlations between apoM and other plasma analytes

Several other analytes were previously tested in this cohort of patients [33], and the correlation between the concentrations of apoM and these analytes were tested (Table 2). The strongest positive correlation was to apoA1 (*r *= 0.52; *P *< 0.001), to total cholesterol (*r *= 0.48; *P *< 0.0001), and to LDL cholesterol (*r *= 0.46; *P *< 0.0001). Lower, but significant correlations to apoB (*r *= 0.28; *P *< 0.0001) and HDL cholesterol (*r *= 0.24; *P *= 0.0014) were observed. Strong negative correlations to the acute-phase markers HBP (heparin-binding protein), procalcitonin, CRP, and IL-6 were observed.

**Table 2:** Correlations between the plasma concentrations of apoM and those of several different analytes tested

Analytes	*r *value	*P *value
Total cholesterol	0.48	< 0.0001
HDL cholesterol	0.24	0.0014
LDL cholesterol	0.46	< 0.0001
ApoA1	0.52	< 0.0001
ApoB	0.28	< 0.0001
Interleukin 6	-0.29	< 0.0001
Procalcitonin	-0.41	< 0.0001
Lactate	-0.35	< 0.0001
C-reactive protein	-0.34	< 0.0001
Heparin-binding protein (HBP)	-0.35	< 0.0001

Spearman rank-correlation test was used.

## Discussion

Sepsis is associated with a strong acute-phase response resulting in pronounced changes in the concentrations of many plasma proteins [17,18,23,26]. This also is true for the lipoproteins, which change in both lipid and protein content [16-19]. HDL is more affected by the acute-phase response than is LDL, with a drastic decrease in the content of cholesterol-esters and changes in protein composition. The concentrations of apoAI and apoAII decrease, and the acute-phase protein SAA increases to the extent that it can become the dominating protein in acute-phase HDL. HDL-associated enzymes PON-1, LCAT, and CETP decrease, and the changes in lipid and plasma composition affect the function of HDL, acute-phase HDL being less efficient in reverse cholesterol transport, in antioxidant, and in antiinflammatory activities. We now add to the list of major protein changes in HDL during the acute phase by describing the drastic decrease in apoM concentrations during the acute-phase response and SIRS, regardless of the causative factor.

Under normal conditions, the major part of apoM is associated with HDL [2,5]. Recently, it was revealed that apoM is the carrier of S1P in HDL [8,9]. S1P bound to apoM provides HDL with a number of vasculoprotective effects, which are mediated by S1P interacting with the S1P_1 _receptor on endothelium [35]. This provides a tonic stimulus to the S1P_1 _receptor, which is important for the formation of endothelial adherens junctions that are crucial for the vascular barrier function [10]. The drastic decrease in apoM plasma concentrations caused by the severe acute-phase response of sepsis presumably results in decreased S1P concentrations and, as a consequence, decreased barrier function. Increased vascular leakage is a hallmark of sepsis, and it is likely that the decrease in S1P-associated apoM would contribute to this. ApoM has also been shown to have antioxidant properties, protecting LDL against oxidative injury [5,36]. This function of apoM is likely impaired during the sepsis, which would add to the decreased antioxidative capacity of HDL that is additionally caused by the decrease in PON-1 levels [16,19].

The low plasma concentrations of apoM during sepsis are either the result of decreased transcription of the *apoM *gene or of the shortened half-life of apoM-containing lipoproteins. ApoM is expressed in the liver and kidney. Systemic inflammation induced by injection of LPS, zymosan, or turpentine into mice results in decreased apoM mRNA levels in both liver and kidney [14]. Moreover, addition of TNF or IL-1 to Hep3B hepatoma cell cultures decreases apoM mRNA levels. These results are consistent with the conclusion that apoM is a negative acute-phase protein, the expression of which decreases during inflammatory conditions. The *apoM *gene transcription is under the control of multiple transcription factors. Hepatocyte nuclear factor 1α (HNF-1α), liver receptor homolog 1 (LRH-1), and Forkhead box A2 increase the expression of the *apoM gene *[37-39]. In this context, it is interesting to note that HNF-1α is downregulated by LPS during the acute-phase response [40]. Moreover, it was recently demonstrated that members of the AP-1 family of proinflammatory transcription factors (c-Jun and JunB) compete with HNF-1α for binding to the same regulatory region in the *apoM *promotor [37]. Increased c-Jun and JunB levels during the acute-phase response thus mediate decreased *apoM *gene transcription and synthesis of apoM. Similar mechanisms account for the decrease in apoA-II and possibly other apolipoproteins during the acute-phase response. Taken together, this suggests that the decrease in apoM levels during acute-phase reactions is mostly explained by decreased transcription of the *apoM *gene, in analogy to the actions of the other negative acute-phase apolipoproteins.

Acute-phase proteins are used clinically as markers of disease severity and as prognostic factors [23,26]. Negative acute-phase proteins can be used in a similar manner, even though this is less commonly the case. Several studies have demonstrated that the magnitude of decrease of lipid and apolipoprotein levels has a predictive prognostic value in sepsis. Thus, very low levels of cholesterol fractions (HDL, LDL, and total), apoAI, apoC1, and CETP have been associated with poor survival [27-32]. The pronounced decrease in apoM levels in patients with severe sepsis, and also in those with SIRS without infection, suggests that apoM may be a useful marker for severity of disease and prognosis. In the present study, the mortality rate was low, and the power of the study was not sufficient to elucidate the predictive power of apoM plasma concentrations.

## Conclusions

We find that, in response to severe sepsis and SIRS of other causes, the plasma levels of apoM demonstrate a decrease, which is more pronounced than those of apoAI and apoB. This suggests that the vasculoprotective effects of apoM and its ligand S1P decrease during the acute-phase response. It remains to be elucidated whether apoM and S1P levels have prognostic value and whether changes in apoM levels contribute to the pathogenesis of SIRS and septic shock.

## Key messages

• apoM plasma concentrations are decreased in patients with sepsis, SIRS, and infections compared with controls

• apoM behaves as a negative acute-phase protein

## Abbreviations

ApoA1: apolipoprotein A1; apoB: apolipoprotein B; apoM: apolipoprotein M; APR: acute-phase response; CETP: cholesterol ester transfer protein; CRP: C-reactive protein; ELISA: enzyme-linked immunosorbent assay; HBP: heparin-binding protein; HDL: high-density lipoprotein; IL-1: interleukin 1; IL-6: interleukin 6; LBP: lipopolysaccharide-binding protein; LCAT: lecithin cholesterol acyl transferase; LDL: low-density lipoprotein; LPS: lipopolysaccharide; PAF-AH: platelet-activating factor acetylhydrolase; PLTP: phospholipid transfer protein; PON-1: paraoxonase 1; SAA: serum amyloid A protein; S1P: sphingosine-1-phosphate; SIRS: systemic inflammatory response syndrome; TNF: tumor necrosis factor; VLDL: very low density lipoprotein; WBC: white blood cell count.

## Competing interests

The authors declare that they have no competing interests.

## Authors' contributions

SBK performed the ELISAs and the cholesterol measurements, assisted in analysis of the data, and wrote the first version of the manuscript. AL participated in the design of the clinical study, included and followed up patients, and assisted in analysis of the data and preparation of the manuscript. PÅ participated in the design of the clinical study, included and followed up patients, and assisted in analysis of the data and preparation of the manuscript. BD initiated the study of apoM in sepsis, supervised the experimental work, analyzed data, and wrote the final manuscript. All authors approved the final version of the manuscript.
